# Teleworking Is Significantly Associated with Anxiety Symptoms and Sleep Disturbances among Paid Workers in the COVID-19 Era

**DOI:** 10.3390/ijerph20021488

**Published:** 2023-01-13

**Authors:** Minji Kim, Inho Park, Hyojin An, Byungyoon Yun, Jin-Ha Yoon

**Affiliations:** 1 Yonsei University College of Medicine, 50, Yonsei-ro, Seodaemun-gu, Seoul 03722, Republic of Korea; 2 Department of Preventive Medicine, Yonsei University College of Medicine, 50, Yonsei-ro, Seodaemun-gu, Seoul 03722, Republic of Korea; 3 The Institute for Occupational Health, Yonsei University College of Medicine, 50, Yonsei-ro, Seodaemun-gu, Seoul 03722, Republic of Korea

**Keywords:** telework, anxiety symptom, sleep disturbance, COVID-19

## Abstract

Due to social distancing during COVID-19, teleworking has spread in Korea. Accordingly, the effects of teleworking on physical and mental health have emerged. We aim to determine the association between teleworking and mental health, including anxiety symptoms and sleep disturbance, in paid workers. The data of paid workers from the Sixth Korean Working Conditions Survey, collected between October 2020 and April 2021, were analyzed. Gender stratification analysis and propensity score matching were performed for variables relevant to sociodemographic and occupational characteristics. Adjusted odds ratios (AORs) and 95% confidence intervals (CIs) for each sex were analyzed using multivariable logistic regression, adjusting for sociodemographic and occupational characteristics. Among 28,633 participants, analyses were performed for anxiety symptoms (teleworkers vs. non-teleworkers; men: 12.1% vs. 4.9%; women: 13.5% vs. 5.3%) and sleep disturbance (men: 33.6% vs. 21.3%; women: 39.7% vs. 25.3%). In male teleworkers, the AORs for anxiety symptoms and sleep disturbance were 1.86 (95% CI: 1.14–3.04) and 1.52 (95% CI: 1.10–2.11), respectively. In female teleworkers, the AORs for anxiety symptoms and sleep disturbance were 1.66 (95% CI: 1.13–2.43) and 1.65 (95% CI: 1.28–2.14), respectively. Our results emphasize the importance of mental health and the need for continuous education and care for teleworkers, given the rapid increase in teleworking.

## 1. Introduction

Telework was first used in the 1970s to define work as the performance of work itself rather than as a place of work from a non-traditional point of view [1]. Although there are several definitions of the term, the common denominator is that the office is not the only place where work can be done and that information technology is a means of work [2]. Telework has emerged as a new way of working in numerous countries, companies, and industries since the development of information and communication technology and the widespread use of cloud systems [3].

Many companies worldwide have responded to the spread of the pandemic by changing their ways of working. Most countries have implemented social distancing to reduce transmission; in this context, telework has been actively adopted as a solution to continue existing work. In Australia, France, and the UK, approximately half of the workers experienced teleworking during lockdowns in 2020. In Japan, teleworking rates increased from 10% to 28% between December 2019 and May 2020, although a nationwide lockdown was not implemented [4]. By industry, over 50% of employees in highly digitalized industries, including information and communications services and professional, scientific, and technological services, experienced telework during the COVID-19 pandemic, recording the highest rates [4].

Teleworking has several advantages and disadvantages. As an advantage, the flexibility and autonomy of professional task management can be improved by autonomously adjusting and managing schedules at the individual level [5]. In addition, reduced transportation costs and commuting time, consequently reducing commuting stress, make workers prefer telework [6]. However, teleworking results in a lack of development of social relationships with co-workers, especially those working in the office [5]. Humans are social animals that must live and interact with other individuals [7]. In addition, the COVID-19 pandemic has deprived most people of close contact with colleagues, friends, and family [8]. Despite the growing number and availability of digital video platforms that facilitate virtual social interactions, social distancing and isolation impact mental health [8,9].

In this regard, the effect of rapidly spreading telework on workers’ health has received attention [4,10,11]. The lack of interaction among workers is a major disadvantage reported by teleworking employees [5]. Social deprivation and loneliness have negative health effects such as poor sleep quality, increased anxiety symptoms, depression, and increased risk of suicide [12,13,14]. In addition, the COVID-19 pandemic period specifically enforced additional restrictions on teleworkers when establishing alternative social relationships, thereby leading to social isolation and reducing work efficiency [15]. Additionally, teleworkers experience sleeping disturbances and the coexistence of housework and work tasks when the boundary between work and home disappears [11]. Existing studies have focused on the factors related to the deterioration of mental health in teleworkers. Although several studies have reported significant results regarding the specific effect of teleworking on mental health, particularly anxiety and sleep, these studies had limitations in that the subject group was limited to full-time teleworkers in certain countries and that the sample size was small [16]. Additionally, the mental health impact of teleworking is expected to differ between countries owing to differences in the spread of COVID-19 and social distancing policies. Given the lack of studies on the mental health of teleworkers in Korea, a study that reflects the circumstances of Korea is required. As there is a lack of meaningful studies conducted in Korea, we have tried to address the circumstances in Korea.

Therefore, this study is aimed at testing the hypothesis that teleworking during the COVID-19 period has impacted mental health, including anxiety symptoms and sleep disturbance, in wage workers.

## 2. Materials and Methods

### 2.1. Participants and Methods

Data from the Sixth Working Conditions Survey (6th KWCS) conducted by the Institute for Occupational Safety and Health from October 2020 to April 2021 were used. The Working Conditions Survey, first conducted in 2006 (1st KWCS), benchmarks the European Working Environment Survey (EWCS) and the UK Labor Force Survey (LFS). It investigates the overall work environment, including working type, employment type, occupation, working hours, exposure to risk factors, and health effects [17].

**Figure 1:**
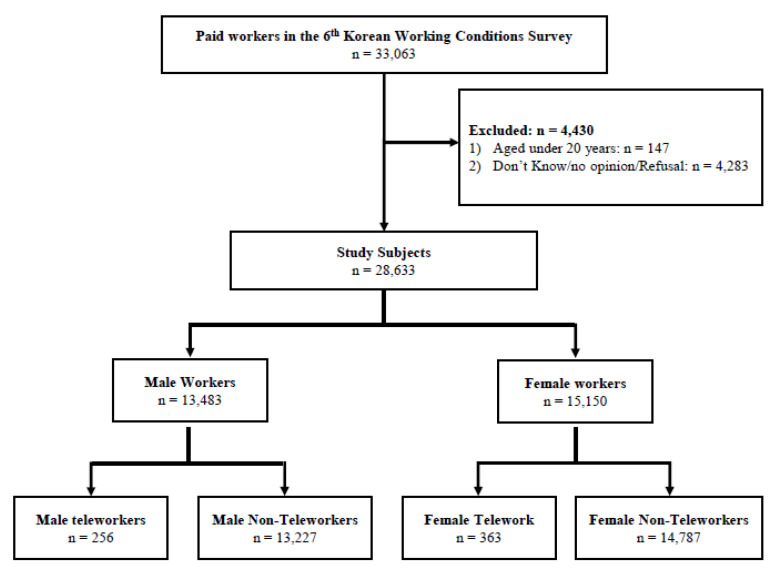
Schematic flow of participant selection process.

Here, we analyze the data of 33,063 paid workers who participated in the recently conducted 6th KWCS in 2021. Wage workers aged ≥20 years were selected for the survey. Participants who did not respond at all or did not respond to major questions such as education, monthly income, working hours, working over 10 h a day, and business size were excluded, and the final analysis included 28,633 people (Figure 1).

### 2.2. Definition of Variables

#### 2.2.1. Main Variables

Teleworking was defined using responses to the question, “In the past year (or after starting your main job, if you have been working for less than a year), how often have you worked at home?” “Always”, “Almost always”, and “Sometimes” were classified as “Yes”, and “Rarely” and “Never” were classified as “No”. Workers who answered “No” were included in the control group.

Anxiety symptoms were defined using the question, “Did you have any of the following health problems (anxiety symptoms) during the past year (if you have been working for less than a year, since you started your main job)?” According to the responses to the question, workers who answered “Yes” were classified as having anxiety symptoms [18,19].

Sleep disturbance was defined using the question, “In the past year (or after starting your main job, if you have been working for less than a year), how often did you have the following problems with sleep?—difficulty falling asleep, waking up frequently during sleep, and tiredness even after waking up”. Workers who answered “Every day”, “Many times a week”, or “Many times a month” in at least one of the abovementioned symptoms in the response were classified as having sleep disturbance, whereas the others were classified under “No sleep disturbance” [20,21].

#### 2.2.2. Covariates

The covariates were based on individual and occupational factors and were considered potential confounding variables. We included demographic characteristics such as age, level of education, and income level as covariates. Age was classified as 20s, 30s, 40s, 50s, and ≥60 years. Level of education was classified as high school graduation or lower and college graduation or higher. Income level was classified by monthly average income into <2 million won, 2–3.99 million won, and ≥4 million won categories.

We included occupational characteristics such as occupational group, employment type of wage workers, weekly working hours, night work, working over 10 h a day, and business size. Occupational groups were classified into four groups: (1) office workers (managers, experts and related workers, office workers); (2) service and sales workers; (3) skilled agricultural, forestry, and fishery workers; and (4) manual workers (technicians and associate professionals, plant and machine operators and assemblers, elementary occupations) according to the 7th Korean Standard Classification of Occupations, which benchmarks the International Standard Classification of Occupations [22]. Wage workers were classified as full-time employees, temporary employees, and day employees. Night work was defined as working for at least 2 h between 10 p.m. and 5 a.m. and was classified as “Yes” or “No” based on the frequency of at least once a month. Working hours included both work preparation time before work hours and clean-up time before leaving work and excluded lunch breaks. We classified working over 10 h a day as “Yes” if its frequency was at least once a month. Weekly working hours were classified as <40 h, 40–51 h, and ≥52 h. Business size was classified based on the number of employees as ≤9, 10–249, and ≥250 according to Organisation for Economic Co-operation and Development (OECD) criteria.

### 2.3. Statistical Analysis

The frequency (%) or mean (standard deviation) of demographic and occupational characteristics stratified by sex and teleworking were estimated. Student’s *t*-test was performed for continuous variables and the chi-square test was used for categorical variables to identify baseline characteristics. Gender stratification analysis was conducted considering differences in the characteristics of mental health between men and women [23]. Additionally, gender stratification analysis and propensity score matching (PSM) were conducted for age, level of education, income level, occupational group, employment type of wage workers, weekly working hours, night work, working over 10 h a day, and business size. The Nearest Neighbor method was used with a caliper of 0.05, and the matching ratio was set to 1:3 between the experimental and control groups. Adjusted odds ratios (AORs) and 95% confidence intervals (CIs) were calculated by multivariate logistic regression models for each sex to identify the association between anxiety symptoms and sleep disturbance with telework. The crude OR was estimated using Model 1. In Model 2, the demographic characteristics, including age, level of education, and income level, were adjusted. Finally, in Model 3, occupational group, employment type of wage workers, weekly working hours, night work, working over 10 h a day, and business size were adjusted in addition to Model 2.

Data were analyzed using the statistical program R version 4.2.1 (R Foundation for Statistical Computing, Vienna, Austria). All data were considered statistically significant when *p*-values were <0.05.

### 2.4. Ethics Statement

The study protocol adhered to the 1975 Declaration of Helsinki’s ethical principles and was approved by the Severance Hospital’s Institutional Review Board (IRB No. 4-2021-1046). Owing to the retrospective nature of the data, informed consent from participants was waived.

## 3. Results

### 3.1. Baseline Characteristics and PSM (Propensity Score Matching) of Study Subjects for Telework

The sixth work environment survey included 13,483 men (47.1%) and 15,150 women (52.9%). The mean age was 45.3 ± 13.7 and 47.4 ± 14.4 years in male and female workers, respectively, and 1.9% (256 people) of male workers and 2.4% (363 people) of female workers said that they had experienced teleworking.

Table 1 summarizes the characteristics of the study participants for teleworking by sex. Teleworking was significantly associated with a high level of education, office worker type, and short working hours. In the stratification analysis, the 40s was the most common age group in both men and women, comprising 31.3% (80 people) of male teleworkers and 27.8% (101 people) of female teleworkers. Regarding the level of education, the majority of teleworkers were college graduates or more highly educated, accounting for 80.9% (207 people) of male teleworkers and 66.7% (242 people) of female teleworkers. Among male teleworkers, salaries of ≥4 million won accounted for 43.0%, whereas it was 11.9% for female teleworkers. Regarding the occupational group, both sexes had the highest percentage of office workers at 72.3% (185 people) and 62.3% (226 people), respectively.

Regarding weekly working hours, 40–51 h accounted for the highest percentage in both men and women at 80.5% (206 people) and 60.3% (219 people), and ≥52 h had the lowest percentage at 7.0% (18 people) and 3.9% (14 people), respectively. Regarding daily working time, 25.0% (64 people) of men and 11.0% (40 people) of women worked for >10 h. Regarding night work, 14.1% (36 people) of men and 11.3% (41 people) of women answered “Yes”, and the proportion was not statistically significant in men (*p* = 0.297).

Regarding the mental health of teleworkers, 12.1% (31 people) and 13.5% (49 people) of male and female workers complained of anxiety symptoms, respectively, and sleep disturbance was observed in 33.6% (86) and 39.7% (144) of patients, respectively.

**Figure 2:**
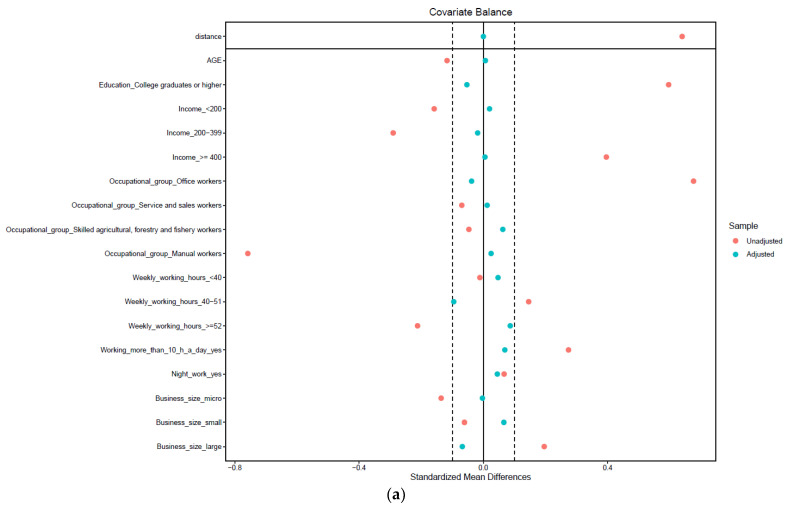

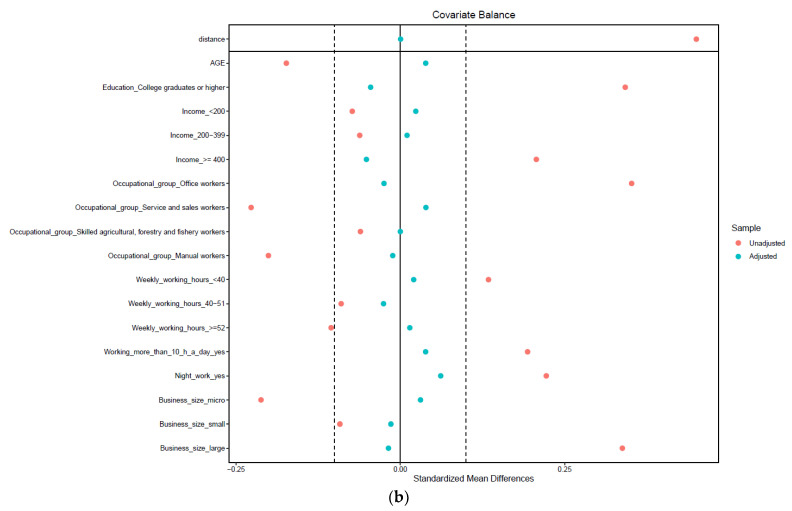
Standardized mean differences in covariates used in propensity score matching. (**a**) Male workers. (**b**) Female workers.

We performed PSM (propensity score matching) for each gender stratum using age, level of education, income level, occupational group, weekly working hours, night work, working over 10 h a day, and business size (Table 2 and Figure 2). After PSM, there was no statistically significant difference in variables according to the status of telework in both men and women.

### 3.2. Relation between Telecommuting Workers’ Anxiety Symptoms and Sleep Disorders by Gender Stratification Analysis

Table 2 shows the results of the gender stratification analysis of telecommuting workers’ anxiety symptoms and sleep disturbance. In the case of male teleworkers, they experienced more problems, including anxiety symptoms (12.1% vs. 6.7%) and sleep disturbance (33.6% vs. 24.4%), than those who did not work from home. In the case of female teleworkers, they experienced more problems, including anxiety symptoms (13.3% vs. 8.6%) and sleep disturbance (39.4% vs. 28.2%), than those who did not work from home.

To determine the association of teleworking with anxiety symptoms and sleep disturbance among male and female workers, Table 3 and Table 4 show the adjusted odds ratios (AORs) and 95% CIs analyzed using multivariate logistic regression.

From the results of Model 3 in the multivariate logistic regression analysis, the AORs (95% CI) of male teleworkers compared with those who did not work from home were 1.86 (95% CI: 1.14–3.04) for anxiety symptoms and 1.52 (95% CI: 1.10–2.11) for sleep disturbance, whereas the AORs (95% CI) of female teleworkers compared with those who did not work from home were 1.66 (95% CI: 1.13–2.43) for anxiety symptoms and 1.65 (95% CI: 1.28–2.14) for sleep disturbance.

## 4. Discussion

This study has investigated the significance of the associations between teleworking, anxiety symptoms, and sleep disturbance. The results remain statistically significant after adjusting for other factors, such as age, education and income level, occupational group, weekly working hours, night work, working over 10 h a day, and business size.

The anxiety symptoms among teleworkers are probably related to the social characteristics of having fewer opportunities to interact with people around them due to working from home in the social distancing situation caused by the COVID-19 pandemic. As mentioned above, social isolation is a proven risk factor affecting mental health, leading to increased anxiety and depressive symptoms [12,13,14]. These results were also presented in a study conducted on teleworkers during the COVID-19 period [24]. In previous studies, the occurrence of isolation and loneliness among teleworkers was significantly higher than that among non-teleworkers [25], which might have resulted in negative emotions such as anxiety symptoms in teleworkers. In addition, difficulties in adapting to rapid social change based on information and communication technology may also cause anxiety. Technostress, a human mental disorder in the era of office automation, affects both work and private life [26]. It is associated with decreased job and life satisfaction and productivity and has often been associated with psychological and behavioral disorders [26].

The teleworking environment increases the risk of obesity and osteoarthritis due to the maintenance of a constant sitting posture without regular breaks and decreasing physical activity [27]. A decrease in physical activity increases the risk of sleep disturbance, although the relationship between physical activity and sleep remains unclear [28]. In addition, the lack of clear separation between personal and family spaces and working space leads to increased stress and the loss of social and occupational regularity, which, in turn, lead to impaired sleep cycle control and sleep quality [29]. Meanwhile, in a meta-analysis, Alvaro et al. suggested that insomnia and sleep quality are bidirectionally related to anxiety, depression, and depression/anxiety [30]. Therefore, anxiety among teleworkers may have affected their sleep. In addition, the higher the exposure to information and communications technology, the lower the quantity, duration, and quality of sleep [31].

The association between anxiety symptoms and sleep disturbance was more prominent among female teleworkers than male teleworkers. Owing to the COVID-19 pandemic, schools conducted remote classes, and as the frequency of all family members going out decreased, homes were transformed from a sleeping space into a place for work, school, and leisure [32]. Time spent in the home residence is a factor that blurs the boundary between work and home with respect to the number of household chores [10,32]. Therefore, due to the blurred boundary between work and home and the increase in chores, teleworkers find it difficult to concentrate on their work and devote sufficient time to work due to housework. In addition, this is more pronounced among female workers, which may result in them being more involved in housework and more affected by increased housework than male workers [33,34].

This large-scale study has analyzed the data of 33,063 people using the raw data of the 6th KWCS and is representative of Korean workers. In addition, the survey was conducted from October 2020 to April 2021 during a specific period of COVID-19 social distancing. As the stratification analysis was conducted considering sex differences, mental health factors attributed to sex were excluded, and it was confirmed that the effects of each factor might vary depending on sex. Confounding biases that could affect mental health factors were reduced through PSM. To the best of our knowledge, this is the first study on anxiety and sleep related to teleworking using the 6th KWCS. Based on this study, in the future, when telecommuting is activated, periodic depression or anxiety screening tests for telecommuters and subsequent cognitive behavioral treatment programs for risk groups can be applied.

The limitations of this study are as follows. First, as this is a cross-sectional study, it is difficult to elucidate the causal relationship between changes in occupational form due to COVID-19, in mental health due to telework, and in separation between work and family. Second, not all variables that can affect mental health, such as smoking, alcohol use, drug use, and history of psychiatric disorders, were considered due to the lack of information. Moreover, there might be unmeasured confounding factors with positive mental health, such as spending more time with family or less commuting time. To reveal elaborate relationships, person-oriented analysis to identify specific characteristics that affect different levels of mental health will be needed in further study. Third, the accuracy of the test was limited because the participants were asked about their anxiety symptoms and sleep in the form of a self-report questionnaire, although previous studies have used the same method, with anxiety symptoms and sleep disturbance as the dependent variable.

## 5. Conclusions

In this study, the association of teleworking with mental health among wage workers was investigated, and the results highlighted that teleworking, which leads to social isolation in the COVID–19 environment, had a strong correlation with anxiety and sleep disorders. There is a need for mental health management for teleworkers, and education and health management should be conducted as the era of telework will expand due to technological developments in the future.

## Figures and Tables

**Table 1:** Characteristics of male and female participants by telework.

	Male Workers (n = 13,483)			Female Workers (n = 15,150)		
	Telework			Telework		
	No (n = 13,227)	Yes (n = 256)	*p*-Value	No (n = 14,787)	Yes (n = 363)	*p*-Value
	No. (%)	No. (%)		No. (%)	No. (%)	
**Age**	45.3 ± 13.7	43.9 ± 11.9	0.066	47.5 ± 14.4	45.2 ± 13.1	0.001
20–29	1830 (13.8%)	30 (11.7%)		1807 (12.2%)	41 (11.3%)	
30–39	3190 (24.1%)	70 (27.3%)		2857 (19.3%)	95 (26.2%)	
40–49	3322 (25.1%)	80 (31.3%)		3462 (23.4%)	101 (27.9%)	
50–59	2688 (20.3%)	48 (18.8%)		3825 (25.9%)	72 (19.8%)	
≥ 60	2197 (16.6%)	28 (10.9%)		2836 (19.2%)	54 (14.9%)	
**Level of education**			<0.001			<0.001
High school graduation or lower	5634 (42.6%)	49 (19.1%)		7314 (49.5%)	121 (33.3%)	
College graduates or higher	7593 (57.4%)	207 (80.9%)		7473 (50.5%)	242 (66.7%)	
**Income level (Monthly)**			<0.001			<0.001
<2 million won	2284 (17.3%)	31 (12.1%)		6684 (45.2%)	151 (41.6%)	
2–3.99 million won	7851 (59.4%)	115 (44.9%)		7340 (49.6%)	169 (46.6%)	
≥4 million won	3092 (23.4%)	110 (43.0%)		763 (5.2%)	43 (11.9%)	
**Occupational group**			<0.001			<0.001
Office workers	5553 (42.0%)	185 (72.3%)		6684 (45.2%)	226 (62.3%)	
Service and sales workers	1789 (13.5%)	29 (11.3%)		4697 (31.8%)	81 (22.3%)	
Skilled agricultural, forestry, and fishery workers	90 (0.7%)	1 (0.4%)		53 (0.4%)	0 (0.0%)	
Manual workers	5795 (43.8%)	41 (16.0%)		3353 (22.7%)	56 (15.4%)	
**Weekly working hours**			0.029			0.014
<40 h	1702 (12.9%)	32 (12.5%)		4345 (29.4%)	130 (35.8%)	
40–51 h	9879 (74.7%)	206 (80.5%)		9572 (64.7%)	219 (60.3%)	
≥52 h	1646 (12.4%)	18 (7.0%)		870 (5.9%)	14 (3.9%)	
**Working more than 10 h a day**			<0.001			<0.001
No	11,490 (86.9%)	192 (75.0%)		14,054 (95.0%)	323 (89.0%)	
Yes	1737 (13.1%)	64 (25.0%)		733 (5.0%)	40 (11.0%)	
**Night work**			0.297			<0.001
No	11,674 (88.3%)	220 (85.9%)		14,156 (95.7%)	322 (88.7%)	
Yes	1553 (11.7%)	36 (14.1%)		631 (4.3%)	41 (11.3%)	
**Business size (the number of workers)**			0.002			<0.001
≤9	4417 (33.4%)	70 (27.3%)		7014 (47.4%)	135 (37.2%)	
10–249	5925 (44.8%)	107 (41.8%)		6198 (41.9%)	136 (37.5%)	
≥250	2885 (21.8%)	79 (30.9%)		1575 (10.7%)	92 (25.3%)	
**Anxiety symptom**			<0.001			<0.001
No	12,574 (95.1%)	225 (87.9%)		13,997 (94.7%)	314 (86.5%)	
Yes	653 (4.9%)	31 (12.1%)		790 (5.3%)	49 (13.5%)	
**Sleep disturbance**			<0.001			<0.001
No	10,409 (78.7%)	170 (66.4%)		11,041 (74.7%)	219 (60.3%)	
Yes	2818 (21.3%)	86 (33.6%)		3746 (25.3%)	144 (39.7%)	

**Table 2:** Characteristics of male and female participants by telework after propensity score matching.

	Male Workers (n = 1022)			Female Workers (n = 1423)		
	Telework			Telework		
Variables	No (n = 766)	Yes (n = 256)	*p*-Value	No (n = 1063)	Yes (n = 360)	*p*-Value
	No. (%)	No. (%)		No. (%)	No. (%)	
**Age**	43.8 ± 12.1	43.9 ± 11.9	0.996	44.7 ± 13.4	45.2 ± 13.1	0.954
20–29	92 (12.0%)	30 (11.7%)		135 (12.7%)	41 (11.4%)	
30–39	217 (28.3%)	70 (27.3%)		286 (26.9%)	95 (26.4%)	
40–49	231 (30.2%)	80 (31.3%)		284 (26.7%)	99 (27.5%)	
50–59	145 (18.9%)	48 (18.8%)		210 (19.8%)	71 (19.7%)	
≥60	81 (10.6%)	28 (10.9%)		148 (13.9%)	54 (15.0%)	
**Level of education**			0.518			0.523
High school graduation or lower	131 (17.1%)	49 (19.1%)		336 (31.6%)	121 (33.6%)	
College graduates or higher	635 (82.9%)	207 (80.9%)		727 (68.4%)	239 (66.4%)	
**Income level (Monthly)**			0.944			0.850
<2 million won	88 (11.5%)	31 (12.1%)		431 (40.6%)	149 (41.4%)	
2–3.99 million won	352 (46.0%)	115 (44.9%)		496 (46.7%)	169 (46.9%)	
≥4 million won	326 (42.6%)	110 (43.0%)		136 (12.8%)	42 (11.7%)	
**Occupational group**			0.365			0.804
Office workers	566 (73.9%)	185 (72.3%)		669 (62.9%)	223 (61.9%)	
Service and sales workers	84 (11.0%)	29 (11.3%)		222 (20.9%)	81 (22.5%)	
Skilled agricultural, forestry and fishery workers	0 (0.0%)	1 (0.4%)		0 (0.0%)	0 (0.0%)	
Manual workers	116 (15.1%)	41 (16.0%)		0 (0.0%)	0 (0.0%)	
Missing	0 (0.0%)	0 (0.0%)		172 (16.2%)	56 (15.6%)	
**Weekly working hours**			0.294			0.804
<40 h	84 (11.0%)	32 (12.5%)		363 (34.2%)	128 (35.6%)	
40–51 h	645 (84.2%)	206 (80.5%)		666 (62.7%)	219 (60.8%)	
≥52 h	37 (4.8%)	18 (7.0%)		34 (3.2%)	13 (3.6%)	
**Working more than 10 h a day**			0.331			0.317
No	599 (78.2%)	192 (75.0%)		966 (90.9%)	320 (88.9%)	
Yes	167 (21.8%)	64 (25.0%)		97 (9.1%)	40 (11.1%)	
**Night work**			0.525			0.103
No	672 (87.7%)	220 (85.9%)		982 (92.4%)	322 (89.4%)	
Yes	94 (12.3%)	36 (14.1%)		81 (7.6%)	38 (10.6%)	
**Business size (the number of workers)**			0.609			0.897
≤9	211 (27.6%)	70 (27.3%)		385 (36.2%)	135 (37.5%)	
10–249	296 (38.6%)	107 (41.8%)		414 (39.0%)	136 (37.8%)	
≥250	259 (33.8%)	79 (30.9%)		264 (24.8%)	89 (24.7%)	
**Anxiety symptom**			0.008			0.011
No	715 (93.3%)	225 (87.9%)		972 (91.4%)	312 (86.7%)	
Yes	51 (6.7%)	31 (12.1%)		91 (8.6%)	48 (13.3%)	
**Sleep disturbance**			0.005			<0.001
No	579 (75.6%)	170 (66.4%)		763 (71.8%)	218 (60.6%)	
Yes	187 (24.4%)	86 (33.6%)		300 (28.2%)	142 (39.4%)	

**Table 3:** Multivariate logistic regression for anxiety symptoms.

(a) Male Teleworkers			
Variables	Model 1 ^a^	Model 2 ^b^	Model 3 ^c^
	AOR (95% CI)	AOR (95% CI)	AOR (95% CI)
**Anxiety symptoms**	1.93 (1.21–3.09)	1.94 (1.21–3.11)	1.86 (1.14–3.04)
High school graduation or lower		1 (reference)	1 (reference)
College graduates or higher		0.91 (0.45–1.86)	0.87 (0.40–1.92)
**Income level:** <2 million won		1 (reference)	1 (reference)
**Income level:** 2–3.99 million won		0.59 (0.27–1.26)	0.40 (0.16–1.00)
**Income level:** ≥4 million won		0.63 (0.28–1.43)	0.39 (0.14–1.07)
Working hours/week <40 h			1 (reference)
Working hours/week 40–51 h			1.20 (0.46–3.13)
Working hours/week ≥52 h			1.33 (0.38–4.57)
Night work/month (no)			1 (reference)
Night work/month (yes)			1.33 (0.71–2.52)
**(** **b)** ** Female Teleworkers**			
**Variables**	**Model 1 ^a^**	**Model 2 ^b^**	**Model 3 ^c^**
	**A** **OR (95% CI)**	**A** **OR (95% CI)**	**A** **OR (95% CI)**
**Anxiety symptoms**	1.64 (1.13–2.38)	1.66 (1.14–2.41)	1.66 (1.13–2.43)
High school graduation or lower		1 (reference)	1 (reference)
College graduates or higher		1.12 (0.66–1.89)	1.13 (0.63–2.04)
**Income level:** <2 million won		1 (reference)	1 (reference)
**Income level:** 2–3.99 million won		2.06 (1.33–3.20)	1.40 (0.80–2.43)
**Income level:** ≥4 million won		1.18 (0.60–2.34)	0.72 (0.33–1.60)
Working hours/week <40 h			1 (reference)
Working hours/week 40–51 h			1.30 (0.75–2.23)
Working hours/week ≥52 h			1.07 (0.38–2.96)
Night work/month (no)			1 (reference)
Night work/month (yes)			1.00 (0.53–1.87)

^a^ Model 1: crude odds ratio; ^b^ Model 2: Model 1 + adjusted for age, education, and income level; ^c^ Model 3: Model 2 + adjusted for occupational group, weekly working hours, night work, working over 10 h a day, and business size.

**Table 4:** Multivariate logistic regression for sleep disturbance.

(a) Male Teleworkers			
Variables	Model 1 ^a^	Model 2 ^b^	Model 3 ^c^
	AOR (95% CI)	AOR (95% CI)	AOR (95% CI)
**Sleep disturbance**	1.57 (1.15–2.13)	1.56 (1.15–2.13)	1.52 (1.10–2.11)
High school graduation or lower		1 (reference)	1 (reference)
College graduates or higher		0.67 (0.44–1.02)	0.74 (0.45–1.20)
**Income level:** <2 million won		1 (reference)	1 (reference)
**Income level:** 2–3.99 million won		0.80 (0.49–1.31)	0.62 (0.33–1.14)
**Income level:** ≥4 million won		0.71 (0.42–1.20)	0.52 (0.26–1.01)
Working hours/week <40 h			1 (reference)
Working hours/week 40–51 h			0.90 (0.50–1.60)
Working hours/week ≥52 h			2.49 (1.12–5.53)
Night work/month (no)			1 (reference)
Night work/month (yes)			1.62 (1.03–2.54)
**(** **b)** ** Female Teleworkers**			
**Variables**	**Model 1 ^a^**	**Model 2 ^b^**	**Model 3 ^c^**
	**A** **OR (95% CI)**	**A** **OR (95% CI)**	**A** **OR (95% CI)**
**Sleep disturbance**	1.66 (1.29–2.13)	1.67 (1.30–2.14)	1.65 (1.28–2.14)
High school graduation or lower		1 (reference)	1 (reference)
College graduates or higher		0.85 (0.61–1.17)	0.84 (0.58–1.19)
**Income level:** <2 million won		1 (reference)	1 (reference)
**Income level:** 2–3.99 million won		1.19 (0.91–1.56)	0.95 (0.67–1.33)
**Income level:** ≥4 million won		1.64 (1.11–2.42)	1.18 (0.74–1.88)
Working hours/week <40 h			1 (reference)
Working hours/week 40–51 h			1.01 (0.73–1.39)
Working hours/week ≥52 h			1.10 (0.54–2.20)
Night work/month (no)			1 (reference)
Night work/month (yes)			1.64 (1.06–2.54)

^a^ Model 1: crude odds ratio; ^b^ Model 2: Model 1 + adjusted for age, education, and income level; ^c^ Model 3: Model 2 + adjusted for occupational group, weekly working hours, night work, working over 10 h a day, and business size.

## Data Availability

This study used publicly accessible information from OSHRI (Korea Occupational Safety and Health Research Institute, https://oshri.kosha.or.kr/oshri/index.do) (accessed on 10 February 2022) in Korea. All data are available from the following URL using a request form: https://oshri.kosha.or.kr/oshri/researchField/introduction.do (accessed on 10 February 2022).
